# The Inhibitory Impact of a Co-Assembly Gel with Natural Carrier-Free Binary Small Molecules, as Used in Traditional Chinese Medicine, on the Viability of SW1990 Cells

**DOI:** 10.3390/gels10090569

**Published:** 2024-08-31

**Authors:** Xueqiang Nie, Sifan Liu, Qiongxue Huang, Haifeng Wu, Qingxia Zheng, Xudong Xu, Bowen Li, Guoxu Ma, Xiaolei Zhou, Shuchen Liu, Weijuan Gao

**Affiliations:** 1 Key Laboratory of Bioactive Substances and Resource Utilization of Chinese Herbal Medicine, Ministry of Education, Institute of Medicinal Plant Development, Peking Union Medical College and Chinese Academy of Medical Sciences, Beijing 100193, China; niexueqiang0220@163.com (X.N.); hqxue@outlook.com (Q.H.); hfwu@implad.ac.cn (H.W.); zhengqingxia916@126.com (Q.Z.); xdxu@implad.ac.cn (X.X.); mgxfl8785@163.com (G.M.); 2 Department of Pharmaceutical Science, Beijing Institute of Radiation Medicine, Beijing 100850, China; 18146539471@163.com; 3 Department of Pathophysiology, Hebei University of Chinese Medicine, Shijiazhuang 050000, China; libowen.1@163.com; 4 Guangxi Botanical Garden of Medicinal Plants, Nanning 530023, China; zhouxl@gxyyzwy.com

**Keywords:** co-assembly, deacetylasperulosidic acid (DAA), gel, puerarin (PUE), traditional Chinese medicine (TCM)

## Abstract

Chinese herbs are a huge treasure trove of natural products and an important source of many active molecules. The theory of traditional Chinese medicine compatibility (TCMC) is widely applied in clinical practice, but its mechanism is still ambiguous. This study aims to open a new window for this predicament by studying the interaction between the main active ingredients from a drug pair. Carrier-free assembly of natural products improves the shortcomings of traditional nanodelivery systems and opens a new path for the development of new nanomaterials. The drug pair “*Pueraria* and *Hedyotis diffusa*” has been commonly used in clinical practice, with a predominant therapeutic effect. This study is devoted to the study of the binary small molecule co-assembly of the main active molecules from the drug pair. In this study, we introduce a carrier-free composite gel, formed by the co-assembly of puerarin (PUE) and deacetylasperulosidic acid (DAA) via non-covalent bonds including π–π packing, intermolecular hydrogen bonding, and C=O π interactions. With a strain point 7-fold higher than that of P gel, the P − D gel exhibited favorable rheological properties. The survival rate of SW1990 cells in the P − D group was only 21.39% when the concentration of administration reached 200 μM. It thus demonstrated activity in inhibiting SW1990 cells’ survival, suggesting potential in combating pancreatic cancer. Furthermore, this research offers a valuable concept for enhancing the mechanical properties and bioactivity of hydrogel materials through the utilization of a multi-component natural small molecule co-assembly approach. More importantly, this provides new ideas and methods for the treatment of pancreatic cancer and the analysis of traditional Chinese medicine compatibility theory.

## 1. Introduction

Renowned for the theory of “preventive treatment of disease”, traditional Chinese medicine (TCM) plays a vital role in preventing disease, alleviating symptoms, and preventing recurrence [1]. Each traditional Chinese medicine decoction is made up of a vast array of tiny natural product molecules [2]. Due to their rich chemical structures, varieties of biological activities, and low biological toxicity, the chemical components of TCM are significant in clinical usage [3]. The interactions between the active components of TCM widely exist in the process of TCM compatibility [4]. Elucidating the interactions between active ingredients can help us understand the mechanism by which Chinese medicine works, but only a few people have tried to explain it clearly. This study focuses on the herb pair “*Pueraria lobata* and *Hedyotis diffusa*”, from “Chai-Ge-Lan-Cao-Tang Decoction” [5] and “Compound Ge-Gen Decoction” [6], which has been recorded as having the effect of clearing away heat and toxic material and the function of relieving throat and reducing swelling.

Supramolecular assembly natural drug hydrogels formed without any structure modification have shown great promise in many fields, because of their excellent biocompatibility, good mechanical properties, and convenient environmental protection [7]. A large number of studies have described that the main mechanism for the formation of supramolecular hydrogels is the ordered rearrangement between molecules caused by non-covalent bonds (electrostatic forces, hydrophobic interactions, hydrogen bonding, and π–π interactions) [8]. So far, many researchers have studied the co-assembly process of binary small molecules. For example, baicalin and sanguinarine assemble to form a gel exhibiting synergistic effects [9], and similar phenomena occur with oleanolic acid and choline [10], and resveratrol and gallic acid [11]. Berberine forms nanoparticles with small molecules such as chrysin [12], hesperetin [13], rhein [14], curcumin [15], and cinnamic acid [16]. Although rapid progress has been made in this field in recent years, it is still impossible to accurately explain the material basis of the action of TCM decoctions.

Puerarin (PUE), an isoflavone derivative extracted from *Pueraria lobata*, exhibits diverse biological effects including blood pressure reduction [17], wound infection treatment [18], anti-inflammatory properties [19], anticancer potential [20], antiviral properties [21], diabetes management [22], cardiovascular and cerebrovascular disease prevention [23], and anti-anxiety effects [24]. Deacetylasperulosidic acid (DAA) is a significant constituent of *Hedyotis alba* and *Fermented Morinda citrifolia* L. (*Noni*). Current research on DAA primarily focuses on its botanical origin and pharmacological activities [25], with its anti-inflammatory [26], antioxidant [27], and anti-obesity properties [28]. Despite its numerous pharmacological activities, the clinical utility of PUE is constrained by its poor solubility and bioavailability [29]. Presently, researchers have devised puerarin hydrogels [30], clathrates [31], nanoparticles [32], and other formulations, primarily emphasizing the interaction with polymers, coating, or covalent linkage. Nonetheless, a safe and easily degradable hydrogel consisting of only small molecules aimed at enhancing puerarin solubility still remains unreported.

As per the literature findings, the gel formed solely by puerarin exhibits drawbacks such as inadequate mechanical strength and limited flexibility [33]. Inspired by the compatibility of Chinese medicine, our study first introduced DAA, known for its excellent solubility, into PUE for the first time to enhance the physical characteristics of the resulting hydrogels and expand their pharmacological activities. In order to have a better understanding of the interaction between the two molecules, the micromorphology of hydrogels was observed by SEM. Subsequently, the mechanical properties and viscoelasticity of the hydrogels were further tested by rheological and microrheological techniques. The gelation mechanism of gels was hypothesized by using XRD, UV, IR, and NMR techniques and subsequently validated and visualized through molecular dynamics simulation. Finally, the function of the gel to inhibit SW1990 cell viability was explored by the MTT method. This research offers valuable insights and information for the development of safe, efficient, and biologically active hydrogel materials composed of natural small molecules through a multi-component co-assembly approach. At the same time, it also provides a new idea for the analysis of the theory of TCM compatibility and the treatment of pancreatic cancer.

## 2. Results and Discussions

### 2.1. Gelation and Morphological Characterization of Hydrogels

**Figure 1:**
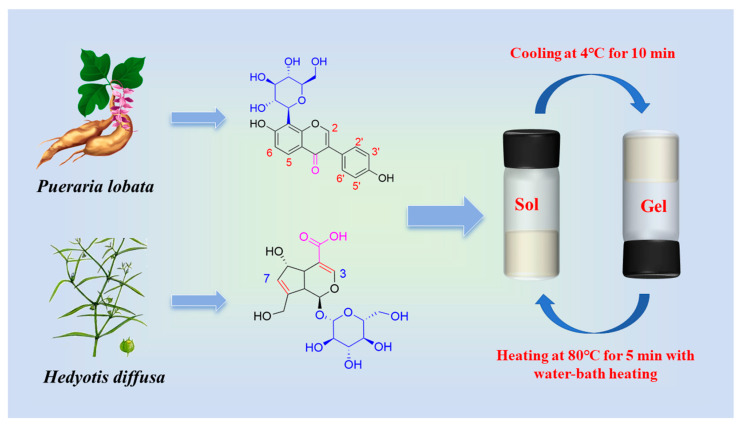
Schematic diagram of chemical structure of PUE and DAA and sol-gel transition of P − D gel.

This research involved the preparation of puerarin self-assembled hydrogels (P gel) and co-assembled hydrogels of puerarin and deacetylasperulosidic acid (P − D gel) through a process of heating followed by cooling. DAA exhibits excellent solubility, readily dissolving without requiring heat, whereas PUE necessitates heating for dissolution acceleration. Gelation in both systems is achieved via a methodology involving heating and subsequent cooling, with the capability to revert the gel to a solution state through reheating (Figure 1). This green and safe process eliminates the need for additional substances or extra steps, relying solely on temperature modulation for effectuation.

**Figure 2:**
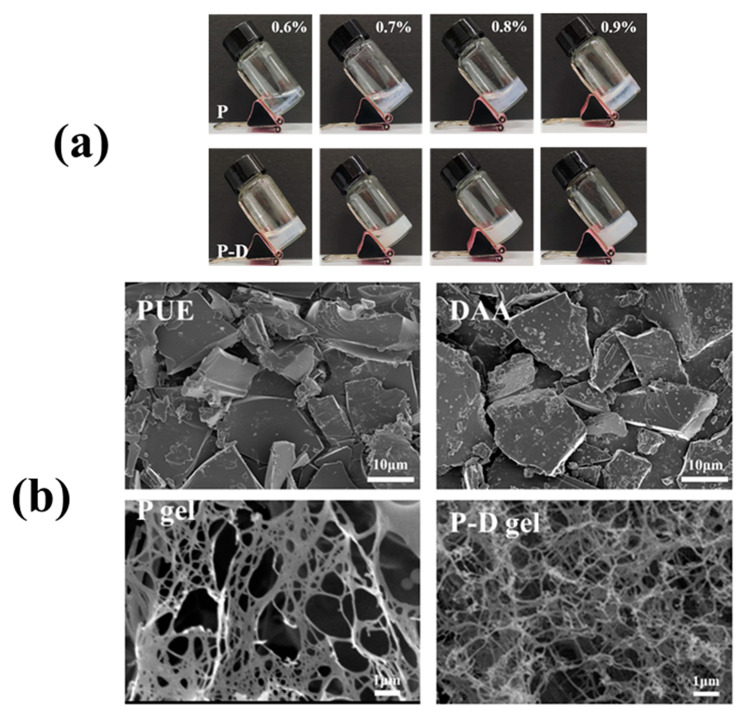
The morphology characteristics of P gel and P − D gel. (**a**) The visual appearance of P and P − D with different concentrations.(**b**) The SEM analysis of the raw material powder of PUE and DAA and the freeze-drying powder of the P gel and P − D gel.

Following this, we examined the observable morphology of P − D gel at an equivalent PUE concentration for comparison, and investigated the critical gelation concentrations of both gels. Figure 2a demonstrates that at a PUE concentration of 0.9%, the 1:1 mixture of PUE and DAA forms a stable and homogeneous milky gel within 5 min, while the monomeric PUE group exhibits a non-flowing solid-like state with significant unevenness, primarily resulting in gel formation at the surface and bottom of the sample container, leaving the center in a liquid state. Decreasing the concentration leads to reduced gel formation. Overall, the P − D gel group displays superior advantages in terms of gelation time, uniformity, and minimum gelation concentration compared to the P gel group, indicating the necessity for further investigation into the co-assembly mechanism of PUE and DAA.

To gain deeper insights into the morphological characteristics of hydrogels, we conducted a Cryo-scanning electron microscopy (SEM) analysis on PUE, DAA, P gel, and P − D gel (refer to Figure 2b). The observations revealed that the P and D monomer groups exhibit a random clustering arrangement before gel formation, which then leads to the development of a network structure. Notably, hydrogels formed solely by PUE display an intricate intertwining of ultrafine fibers in an irregular manner. In contrast, the network of fibers in P − D gel is denser, resulting in the formation of smaller pores that enhance the gel’s strength and resistance to external forces [34]. The disparity in appearance does not suffice to determine the distinction in the system, hence, the rheological properties of the two gel groups were examined to conclusively establish the differentiation between them.

### 2.2. Micro-Rheological and Rheological Analysis

**Figure 3:**
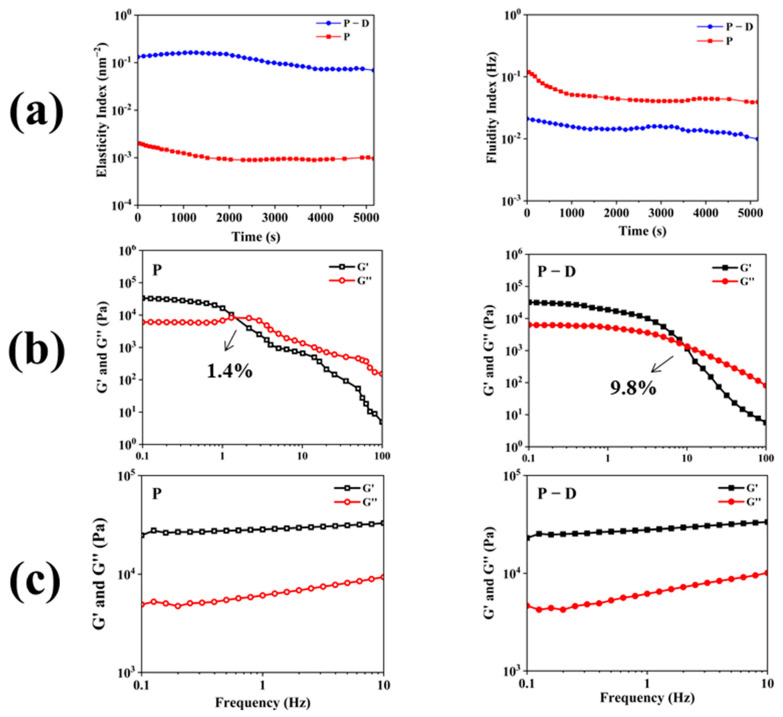
The micro-rheological and rheological analysis of P gel and P − D gel. (**a**) The variations of (left) EI, (right) FI with time at 25 °C. (**b**) Strain-dependent oscillatory shear rheological properties of the P gel (left) and P − D gel (right) at a frequency of 1 Hz (T = 25 °C). (**c**) Frequency-dependent oscillatory shear rheological properties of the P gel (left) and P − D gel (right) at the strain of 0.5% (T = 25 °C).

Micro-rheological tests were conducted on both gels. Figure 3a displays the test results of P gel and P − D gel at the same concentration at room temperature. The data demonstrate that the elasticity index (EI) of P − D gel is significantly higher than that of P gel, indicating a substantial improvement in the elasticity of P − D after co-assembly with DAA. The fluidity index (FI) reflects the sample’s flow capacity under shear forces, and a higher FI value indicates better flow.

Oscillatory shear rheological tests (Figure 3b,c) were further employed to compare the physical property differences between P and P − D gels. As the applied stress force increases, the energy storage modulus (G′) of P gel gradually approaches and intersects with the loss modulus (G″) at 1.4% strain (Figure 3b). This suggests that when the strain exceeds this value, the gel network is destroyed and transformed into a solution state (gel state: G′ > G″, solution state: G′ < G″), confirming the shear thinning behavior of the gel. The yield strain point of P − D gel increased to 9.8% after the addition of DAA, indicating its higher mechanical strength, which is consistent with the microrheological results. Additionally, rotary rheological tests were performed to investigate the viscoelastic properties of the gel. The hydrogel’s oscillating frequency scanning is presented in Figure 3c, ranging from 0.1 to 10 Hz. Both gels were consistently G′ higher than G″ and about eight times higher than the latter, showing their high stability.

### 2.3. Co-Assembled Mechanism of P − D Gel

**Figure 4:**
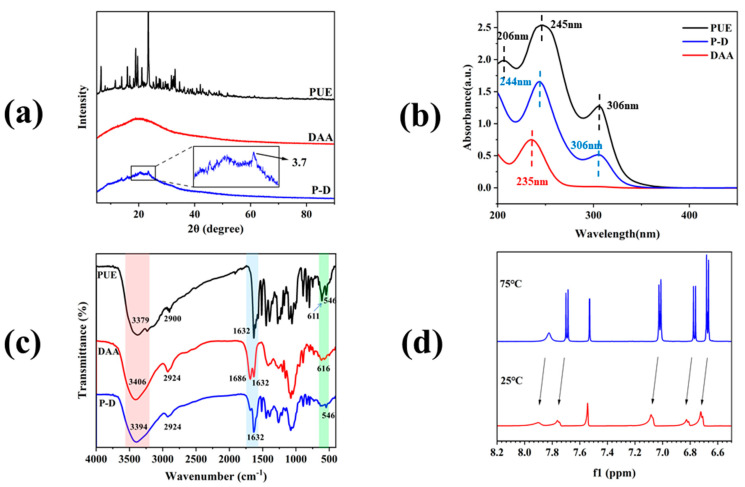
(**a**) XRD results of the monomer of PUE, DAA, and P − D gel; (**b**) UV spectra of the monomer of PUE, DAA, and dried P − D gel; (**c**) FTIR spectra of PUE, DAA, and P − D gel; (**d**) ^1^H NMR spectra of dried P − D gel in D_2_O at 75 °C (top) and 25 °C (bottom).

X-ray diffraction (XRD) has emerged as a crucial tool for investigating the microstructural characteristics of both crystalline and amorphous materials [35]. Smaller grain sizes are associated with reduced diffraction peak intensities, potentially leading to the formation of larger particles upon interaction [36]. In the study, XRD was utilized to assess the crystallinity of free PUE, DAA, and the freeze-dried powders of the P − D complex hydrogel (Figure 4a). Results revealed sharp, well-defined diffraction peaks for free PUE, indicative of its crystalline nature. Conversely, DAA exhibited broad, featureless peaks, suggesting an amorphous structure. The disappearance of crystalline peaks in the P − D gel, resembling those of free PUE, further confirmed its amorphous state and the interaction between the monomers. Moreover, the enhanced bioavailability of PUE in the P − D gel, attributed to the superior solubility of amorphous materials over crystalline counterparts, underscores the significance of this transition from crystalline to amorphous states [37]. In addition, at 23.5°, a small peak of P − D gel was detected and d = 3.7 Å, which is the representative distance of intermolecular π–π interaction, which demonstrated the presence of π–π stacking interactions in the P − D gel.

The structure of the sample was determined by UV-visible spectroscopy (Figure 4b). The results showed that the UV absorption spectra of P − D gel contained the characteristic absorption peaks of PUE and DAA, indicating that the P − D gel was successfully synthesized. At the same time, the position of the characteristic peaks did not change, indicating that the skeleton structure would not be changed after binding. The characteristic peak of DAA is red shifted from 235 nm to 244 nm, which may be due to the π–π interaction [9]. In addition, after the formation of P − D gel, the UV absorbance intensity is significantly reduced compared with PUE, which could be attributed to the steric congestion associated with the supramolecular architecture formed during co-assembly [38].

Utilizing the FTIR technique enables the observation of changes in characteristic peaks at various locations, thereby elucidating the impact of intermolecular interactions on molecular functional groups. This technique serves as a potent tool for unveiling the driving force of hydrogen bonds in the formation of molecular complexes [39]. In the infrared spectrum (Figure 4c) of the PUE molecular, the functional characteristic peak of PUE is detected at 3379 cm^−1^ (hydroxyl stretching vibration), 2900 cm^−1^ (C-H stretching vibration), 1632 cm^−1^ (C=O stretching vibration), 1515 cm^−1^, 1447 cm^−1^, 1396 cm^−1^, and approximately 790–890 cm^−1^, which belong to the vibration of the aromatic ring, 1273 cm^−1^ and 1059 cm^−1^ (C-O-C stretching vibration), with 892 cm^−1^ and 611 cm^−1^ representing characteristic glucose peaks [40]. Similar observation on PUE were reported earlier [41]. Regarding DAA, the broad peak at 3406 cm^−1^ is attributed to hydroxyl stretching vibrations, while 2924 cm^−1^ corresponds to C-H stretching. Peaks at 1686 cm^−1^ and 1632 cm^−1^ signify C-O vibrations in the carboxyl and glycoside chains, respectively, with 1417 cm^−1^ indicating C-H bending vibration, 1077 cm^−1^ representing C-O-C stretching vibration, and 893 cm^−1^ and 616 cm^−1^ being characteristic sugar peaks. In the P − D gel, alongside the characteristic peaks of the individual monomers, certain peak positions and intensities remain consistent, with notable shifts resulting from hydroxyl group stretching vibrations and intermolecular hydrogen bonding. The interaction between the carboxyl group of DAA and the sugar group segment of PUE leads to a broad peak at 3394 cm^−1^. At the same time, the intensity of the peak at 1685 cm^−1^ belonging to DAA and the peak at 610 cm^−1^ belonging to the sugar group decreased significantly, which also confirmed our hypothesis. In summary, we conclude that the P − D molecular complex is successfully formed due to the intermolecular forces between PUE and DAA, especially the intermolecular hydrogen bond.

In general, Nuclear Magnetic Resonance Spectroscopy (NMR) techniques can help elucidate the co-assembly of the gelling process. In this study, the H shift of P − D gel during gelation was observed by variable temperature nuclear magnetic resonance technique. Figure 4d shows the ^1^H NMR spectra of mix solutions at high temperature (75 °C) and hydrogel formed at low temperature (25 °C), with PUE and DAA dissolved in D_2_O. The data describe that the ^1^H NMR chemical shift, which belongs to PUE, changes significantly. In solution state, the aromatic proton signal of PUE occurs at δ = 7.82 (s, 1H, C2-H), 7.69 (dd, C5-H), 7.02 (dd, C2′ 6′-H), 6.77 (dd, C6-H), 6.67 (dd, C3′ 5′-H). In addition, with the decrease of temperature, the solution gradually transforms into a gel state, the proton signal on all aromatic rings shifts to a low field, and the intensity decreases sharply, making it difficult to see the state of peak splitting, which is a strong evidence for the formation of gel. This suggests that the action may be intermolecular via C=O π, and that π–π packing thus transforms the solution into gel state. This type of action has been found in other structures as well [42].

### 2.4. Quantum Chemical Calculation of P − D Gel

**Figure 5:**
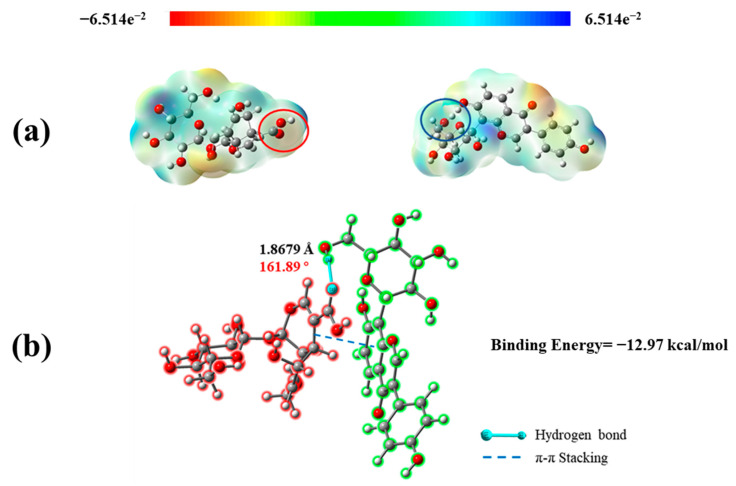
(**a**) Electrostatic potential energy diagram of DAA (left) and PUE (right); (**b**) molecular binding mode diagram. The red structure belongs to the DAA and the green structure belongs to the PUE. The solid blue line represents hydrogen bonds, and the dashed blue line represents π–π interactions.

To clarify the mechanism of the co-assembly of PUE and DAA, quantum chemical calculations of two molecules and molecular complexes were performed using Gaussian 16 software. According to the Hermann–Feynman theorem, non-covalent interacting bonds are intrinsically purely coulombic. The electrostatic potential of molecules can help researchers to preliminarily determine the sites of possible action by different potentials [43]. Figure 5a shows that carbonyl, hydroxyl, and carboxyl groups between two molecules have different degrees of ability to form intermolecular forces. According to Figure 5b, in the binding mode after energy minimization, the binding energy of −12.97 kcal/mol shows the stability of the molecular complex. There is one hydrogen bond between the carbonyl group on DAA and the -CH_2_-OH on the PUE sugar. Furthermore, its bond length is 1.8679 Å, which is far less than the traditional hydrogen bond distance of 3.5 Å [44]. In addition, the double bond in DAA also forms π–π interaction with the benzene ring of PUE. The presence of these intermolecular forces greatly increases the stability of the P − D molecular complex.

### 2.5. Molecular Dynamics Simulation

**Figure 6:**
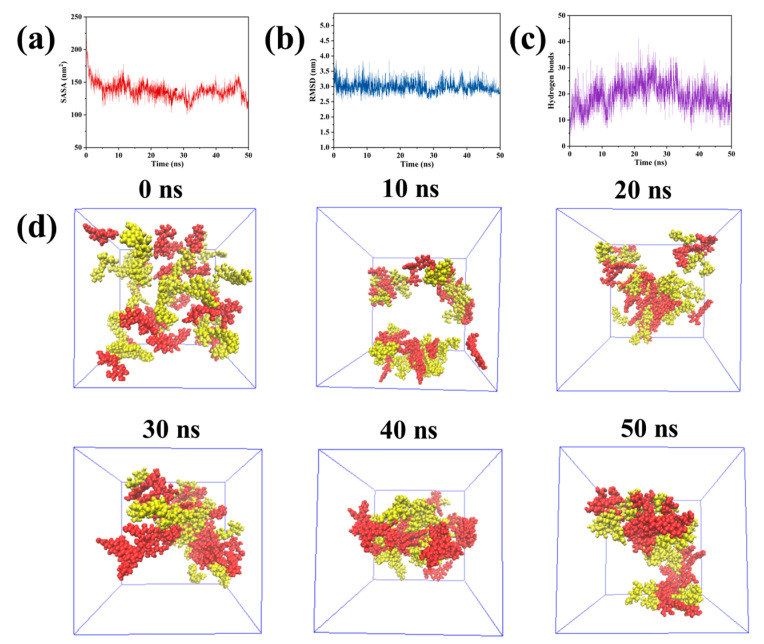
The (**a**) SASA, (**b**) RMSD, (**c**) hydrogen bonds change over time during the co-assembly of simulated P − D molecular complexes. (**d**) The change of molecular conformation with time during the MD simulation of the co-assembly process of PUE–DAA system.

Studies have shown that all-atom molecular dynamics simulations (MDS) have emerged as a key tool to explain the aggregation stage of supramolecular gelation in depth. The method has gained prominence as an approach for comprehending the gelation process [45]. In order to study the co-assembly process of P − D gel and the tightness of the final complex structure, we carried out MDS. Moreover, some parameters can be obtained from the MDS to explain the process of supramolecular gelation. Solvent accessible surface area (SASA) refers to the cumulative surface area of molecules that can interact with the solvent. This parameter is indicative of the molecular–solvent interactions as well as the aggregation behavior among molecules [46]. Adverse interactions between the solvent and the molecules can lead to aggregation, resulting in a reduced SASA. As shown in Figure 6a, there is a notable decrease in SASA between 0 and 5 ns, indicating a swift aggregation of PUE and DAA molecules during this period. The lowest SASA value observed at 30 ns signifies that the system has achieved its maximum aggregation state by this point. The root mean square deviation (RMSD) is a metric for evaluating molecular fluctuations throughout the simulation [47]. Similar trends to the SASA are evident in Figure 6b, confirming that the PUE–DAA composite system attains a relatively stable configuration following 30 ns of simulation. Additionally, the simulation process calculates the number of intermolecular hydrogen bonds, with results depicted in Figure 6c indicating an average of 19.5 hydrogen bonds, peaking between 25 and 30 ns. This finding underscores the significant role that hydrogen bonds play within the P − D molecular complex system. Cluster analysis of the simulation trajectory for the complex system was conducted using the CLUSTER module of Gromacs. Figure 6d shows that all of the molecules in the complex system can aggregate to form a stable cluster of nanofibers as the simulation time continuously accumulates. This is consistent with the microstructure displayed by the SEM.

### 2.6. Study on Antitumor Activity In Vitro

**Figure 7:**
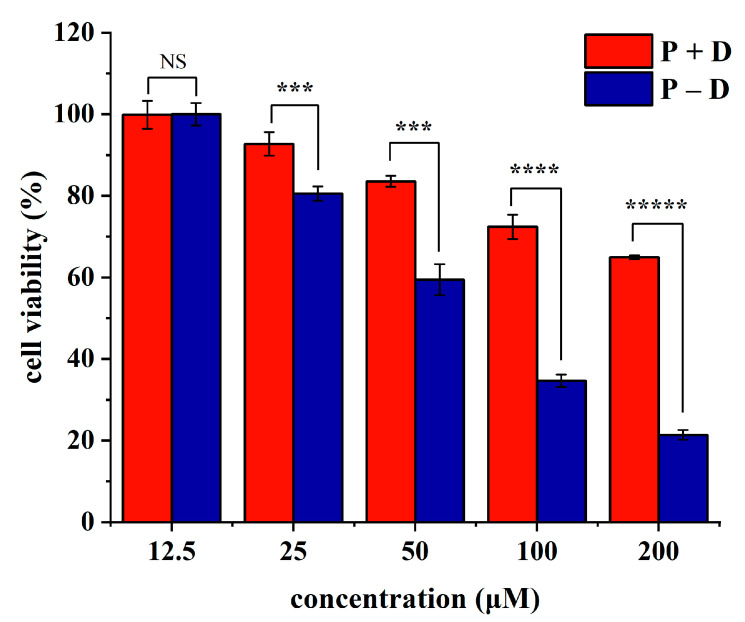
Effect of non-assembled mixture (P + D) and P − D gel on SW1990 cell survival. The viability of human SW1990 pancreatic cancer cells was evaluated through an MTT assay. (NS indicates not significant; *** indicates *p* < 0.001; **** indicates *p* < 0.0001; ***** indicates *p* < 0.00001).

Pancreatic cancer, famous as the “king of cancer”, is a remarkably deadly malignancy characterized by rapid progression, lack of specific therapies, and poor outcomes [48]. The demand for innovative drugs, targets, and treatment modalities is pressing. Research indicates that PUE may exhibit therapeutic potential in pancreatic cancer by inhibiting mTOR-mediated glucose metabolism [49]. Additionally, another important feature of pancreatic cancer is high inflammation [50], and both PUE and DAA have good anti-inflammatory function. Therefore, it is of certain significance to explore the therapeutic effect of P − D gel on pancreatic cancer. Cytotoxicity studies (Figure 7) using MTT assay revealed that treatment with a combination of PUE and DAA (P + D) or P − D gel (P − D) at concentrations up to 12.5 μM did not significantly affect the viability of SW1990 cells. And both groups showed concentration-dependent inhibitory effects on cell viability. Excellent antitumor activity may be related to the synergistic effect of the interacting two small molecules, which may play a role in explaining the material basis of the compatibility of TCM. In addition, compared with P + D, P − D had stronger inhibitory effect on SW1990 cells at the same concentration, which was more obvious at higher concentration. When the concentration reached 100 μM and 200 μM, the survival rate of cells treated with P + D was 72.43% and 64.95%, while the survival rate of cells in P − D group was only 34.66% and 21.39%. Based on this, we found that, as previously reported in the literature, oleanolic acid nanoparticles and nanogels with different elasticity may exhibit different antitumor activities by changing the degree of endocytosis and lysosomal deformation escape without changing the chemical substance [51]. Moreover, the adhesion and sustained release of the gel may also have an impact on its pharmacological activity. Therefore, we speculate that similar to the above conclusions, due to the difference in intermolecular interactions, anti-tumor mechanism of the P + D and the P − D in this study may be different, resulting in a large gap in results. Previous literature has shown that environmentally responsive gels have great potential to treat pancreatic cancer and are often administered in situ [52]. And small-molecule gels show great advantages in cancer treatment because of their excellent biocompatibility and safety. Therefore, preparation of environmentally responsive natural small-molecule gels could be an important therapy for pancreatic cancer in the future.

## 3. Conclusions

In this study, we developed a supramolecular complex composed of PUE and DAA from the drug pair “*Pueraria lobata* and *Hedyotis diffusa*”. Through the change of the peaks in the map of XRD, UV, IR, and NMR, we concluded that the two monomers co-assemble into hydrogel through intermolecular hydrogen bonding, π–π packing, C=O·π, and other forces. Moreover, the above results were verified by quantum chemical calculations and molecular dynamics simulations. As confirmed using SEM images and the rheological performance test, for PUE, the addition of DAA increases the density of molecular aggregates and reduces the pores of the gel network, which can effectively improve the mechanical strength of the gel and thus give the gel better water retention ability. At the same time, the formation of P − D gel also improves the solubility of PUE, which can improve the bioavailability of this insoluble molecule. In addition, the stronger inhibition of the P − D gel on SW1990 cell viability in the MTT test compared with the P + D group suggests that the mechanism by which the drug acts may differ after gel formation. However, this mechanism was not deeply investigated in this study, which may be a potential future research direction. This discovery uses the principle of natural small-molecule co-assembly to prepare hydrogels, which provides a new idea for the treatment of pancreatic cancer. Moreover, it provides insights into the analysis of the theory of the compatibility of TCM.

## 4. Materials and Methods

### 4.1. Reagents, Cell Line, and Materials

In this study, PUE (CAS: 3681-99-0, BR, 98%) was purchased from Shanghai Yuanye Bio-Technology Co., Ltd. (Shanghai, China) and DAA (CAS: 14259-55-3, BR, 98%) was purchased from Chengdu HerbSubstance Co., Ltd. (Chengdu, China). Ultra-pure water was used, and other solvents were provided by the Beijing Chemical plant. The human pancreatic carcinoma cell line, SW1990, was obtained from Cell Resource Center, Peking Union Medical College (PCRC) (Beijing, China).

### 4.2. Preparation of the P Gel and P − D Gel

P gel: 6, 7, 8, and 9 mg of PUE were dispersed in 1 mL of ultra-pure water and heated at 80 °C with water-bath heating for 5 min until completely dissolved. The fully dissolved solution was placed in a refrigerator at 4 °C for 10 min to obtain a stable and evenly distributed gel. The inverted test tube method was used to preliminarily judge the flow behavior of the gel and then determine whether the gel was formed, and a photo was taken as a record. The critical gel concentration is 1.4%.

P − D gel: The preparation method was the same as that of P gel. The difference was that DAA at a molar ratio of 1:1 was mixed with 6, 7, 8 and 9 mg of PUE and completed subsequent manipulation to prepare the P − D gel. The critical gel concentration is 0.9%.

The powder of P gel and P − D gel were prepared by the freeze-drying method.

### 4.3. Morphological Characterization

SEM images of the powder of PUE, DAA, P gel, and P − D gel were recorded using a field-emission scanning electron microscope (JSM-6700F, JEOL, Tokyo, Japan) at 10 mA, 20 kV. The materials used for SEM observation here are the pharmaceutical raw material powder of PUE and DAA, and the lyophilized powder of P gel and P − D gel. Before the observation, the four powders were placed on the sample table, respectively, for gold spraying treatment.

### 4.4. Microrheological Properties Test

An amount of 3 mL of the P gel and P − D gel were prepared in a bottle fitted with an optical rheometer, respectively, using the method described in Section 4.2.

Utilizing the optical rheometer, the long-term stability of P gel and P − D gel were monitored in real-time at room temperature using the Rheolaser MASTER™ system from Formulaction, France. The gels were maintained at 25 °C in the rheometer, and the FI and EI values for both gels were recorded throughout the observation process.

### 4.5. Rheological Properties Test

2 mL of the P gel and P − D gel were prepared in a cylindrical container of 1.5 cm in diameter, respectively, using the method described in Section 4.2.

Rheological investigations were conducted utilizing a rotating rheometer (Kinexus, Germany) equipped with a 40 mm diameter rotor operating at a clearance height of 1.0 mm and a temperature of 25 °C. The oscillatory amplitude sweep was performed at a frequency of 1 Hz, measuring G′ and G″ from 0.1% to 100% strain. A frequency sweep ranging from 0.1 to 10 Hz was carried out with a strain set at 0.5%.

### 4.6. Co-Assembled Mechanism Study

XRD: The powder of PUE, DAA, and the freeze-dried powder of the P − D gel were ground into superfine powder, pressed into thin sheets, and analyzed by using an X-ray diffractometer (X′Pert PRO MPD, Germany) at a range of 5° to 90° and a scanning speed of 4°/min.

UV: The powder of PUE, DAA, and the freeze-dried powder of the P − D gel were dissolved in ultra-pure water to the same concentration, and the UV spectra in the range of 200–800 nm were recorded using UV-3600 (Shimadzu, Kyoto, Japan).

FTIR: The FTIR analysis was carried out utilizing the Nicolet IS10 instrument from Thermo, USA. The measured data were acquired via the potassium bromide (KBr) technique across the spectral range of 4000–400 cm^−1^. The materials used for the experiments were the pharmaceutical raw material powder of PUE and DAA, and the lyophilized powder of P − D gel.

NMR: An amount of 4.16 mg PUE was mixed with 3.9 mg DAA (molar ratio 1:1) and dissolved completely with 500 μL D_2_O, and the ^1^H NMR spectra were obtained using a Bruker AV 600 NMR spectrometer (Bruker, Billerica, MA, USA) at 25 °C (gel) and 75 °C (sol).

### 4.7. Quantum Chemical Calculation

Gaussian 16 software was used to conduct DFT-based calculations, while the chemical compound structures were sourced from the PubChem database. Chem3D software’s MM2 module was utilized to optimize the chemical structure and reduce energy levels, followed by application of the Gaussian 16 suite to optimize the configurations. Structural optimization was performed using the B3LYP functional with the 6-311G(d) basis set with dispersion correction (GD3BJ). 

### 4.8. Molecular Dynamics Simulation

The MDS of P − D gel in pure water was studied by using the Gromacs 2020.06 program. (General Amber force field) GAFF2 all-atomic force field and TIP3P water model were applied. PACKMOL 20.3.5 was used to randomly fill 25 PUEs and 25 DAAs into a 5 × 5 × 5 nm^3^ cube box with a sufficient amount of water molecules to form a simulation system. Before MD simulation, the V-rescale thermostat and Parrinello–Rahman barostat were employed to ensure the equilibrium of temperature and pressure. In order to eliminate the possible spatial stress, the steepest descent approach was utilized for energy minimization within the system. Throughout the simulation, the LINCS algorithm was applied to constrain the hydrogen bond, and the remote electrostatic interaction was treated by the particle grid Ewald method. The above system was simulated by 50 ns, and the SASA, RMSD, hydrogen bond number, and simulation trajectory of the model molecular complex were predicted. The conformational formation simulation track was saved every 10 ns and the simulation results were visualized using the Gromacs embedded program and VMD.4.9. Cell Test.

### 4.9. Cell Test

#### 4.9.1. Cell and Culture Conditions

SW1990 cells were cultured in Dulbecco’s modified Eagle’s medium (DMEM) culture medium containing 10% fetal bovine serum (FBS) and 1% penicillin–streptomycin at 37 °C, in a culture incubator with 95% air and 5% CO_2_.

#### 4.9.2. Cytotoxicity Assay

The 1.2 × 10^4^ healthy SW1990 cells were seeded onto 96-well plates and cultured in a 5% CO_2_ incubator at 37 °C for 24 h. Subsequently, P + D and P − D (12.5, 25, 50, 100, 200 μM) were added for another 24 h, followed by the assessment of cell viability.

## Data Availability

Data available on request from the authors.
